# Supplementary material for: Access to scientific literature by the conservation community
Source: PeerJ. 2020 Jul 9;8:e9404. doi: 10.7717/peerj.9404 (PMC7354838; doi:10.7717/peerj.9404)
Supplement: Supplemental Information 1 [file peerj-08-9404-s001.pdf]

IUCN Access to Scientific Literature Survey

Thank you for responding to this survey, administered by the IUCN HQ Library, about your access to the scientific literature.

The purpose of this survey is to understand the importance of access to scientific literature for the IUCN Secretariat, Commissions, and Membership, to guide the IUCN Science & Knowledge Unit – and specifically the IUCN HQ Library – in prioritising effort to better support you. To do this we need to understand your actual and needed levels and means of access to the scientific literature. Throughout the survey, we define the “scientific literature” as peer-reviewed scientific journals plus technical books.

The survey should take you about 5-7 minutes to complete, and is anonymous. You will have the opportunity to leave your contact information at the end, should you wish to do so.

1. To which component(s) of IUCN do you belong? Tick all that apply.

- [ ] IUCN State Member
- [ ] IUCN Government Agency Member
- [ ] IUCN International NGO Member
- [ ] IUCN National NGO Member
- [ ] IUCN Affiliate Member
- [ ] IUCN CEC member
- [ ] IUCN CEESP member
- [ ] IUCN CEM member
- [ ] IUCN SSC member
- [ ] IUCN WCEL member
- [ ] IUCN WCPA member
- [ ] IUCN Secretariat staff
- [ ] Other (please specify)

2. In which country/territory are you based?

3. Gender

- ( ) Female
- ( ) Male

* 4. How frequently should you be consulting scientific literature to carry out your IUCN-related work?

Never	Infrequently	Sometimes (once a month)	Frequently (once a week)	Very frequently (daily)
○	○	○	○	○

* 5. How easy is it for you currently to obtain the scientific literature you need to carry out your IUCN-related work?

Not at all easy	Not easy	Easy	Very easy
◯	◯	◯	◯

Please explain (if necessary).

* 6. How important is it for your IUCN-related work to have easy access to scientific literature?

Not at all important	Somewhat important	Very important	Essential
◯	◯	◯	◯

Feel free to explain further.

7. Which one scientific journal would have the largest impact on your IUCN-related work if you could obtain easy access to it?

8. In what format do you prefer to read scientific literature?

	I prefer reading on a screen	I prefer printing out to read	I prefer the original hard copy
Journal articles	◯	◯	◯
Books	◯	◯	◯

Other (please specify)

* 9. Do you have institutional access to scientific literature online (e.g. through affiliation with a university or other organization)?

- ◯ Yes
- ◯ No

* 10. How frequently do you use the following means to access scientific literature for your IUCN-related work?

	Never or not available	Infrequently	Sometimes (once a month)	Frequently (once a week)	Very frequently (daily)
I use the library of my own institution.	◯	◯	◯	◯	◯
I visit a local library (public, academic, etc.) to read scientific literature in print.	◯	◯	◯	◯	◯
I use my institutional access to scientific literature online (e.g. through affiliation with a university or other organization).	◯	◯	◯	◯	◯
I request journal articles from the IUCN Librarian.	◯	◯	◯	◯	◯
I request journal articles from the author.	◯	◯	◯	◯	◯
I access scientific literature through my own personal subscription to individual journals.	◯	◯	◯	◯	◯
I ask a friend or colleague whom I know has access to scientific literature online.	◯	◯	◯	◯	◯
I access whatever I can find online for free (via Google Scholar, open-access journals, ResearchGate, etc.)	◯	◯	◯	◯	◯

11. If you had institutional access to scientific literature online, how often would you use this for your IUCN-related work?

Never	Infrequently	Sometimes (once a month)	Frequently (once a week)	Very frequently (daily)
○	○	○	○	○

* 12. What effect would institutional access to scientific literature online have on the quality of your IUCN-related work?

No effect at all	Slight positive effect	Moderate positive effect	Great positive effect
○	○	○	○

Feel free to explain further.

* 13. What impact does lack of institutional access to scientific literature online have on your IUCN-related work?

No impact at all	Slight negative impact	Moderate negative impact	Great negative impact
○	○	○	○

Feel free to explain further.

14. Do you have any other comments, questions, or concerns?

15. If you don't mind being contacted in the future (e.g. to further elaborate on your answers or to participate in a focus group), please leave your contact information below.

Name

Email Address
