# Supplementary material for: Access to scientific literature by the conservation community
Source: PeerJ. 2020 Jul 9;8:e9404. doi: 10.7717/peerj.9404 (PMC7354838; doi:10.7717/peerj.9404)
Supplement: Supplemental Information 2 [file peerj-08-9404-s002.pdf]

Encuesta UICN sobre el acceso a la literatura científica

Gracias de antemano por responder a esta encuesta, gestionada por la Biblioteca de la sede de la UICN, sobre su acceso a la literatura científica.

El propósito de este estudio es comprender la importancia del acceso a literatura científica para la Secretaría, las Comisiones y los Miembros de la UICN, para guiar a la Unidad de Ciencia y Conocimiento de la UICN (Science and Knowledge Unit) -- y, específicamente, a la Biblioteca de la sede de la UICN -- en vistas a mejorar nuestro apoyo en este tema. Para ello necesitamos averiguar cuál es el nivel y forma de acceder a la literatura científica que precisan. A lo largo de esta encuesta, definimos "literatura científica" como las revistas científicas revisadas por pares, además de libros técnicos.

La encuesta dura aproximadamente 5-7 minutos y es anónima. Tendrá la oportunidad de dejar su información de contacto al final, en caso de que desee hacerlo.

1. ¿A cuál de los componentes de la UICN pertenece usted? Marque todo lo que corresponda.

- [ ] Estado Miembro
- [ ] Agencia gubernamental Miembro
- [ ] ONG internacional Miembro
- [ ] ONG nacional Miembro
- [ ] Miembro afiliado
- [ ] Miembro de la CEC
- [ ] Miembro de la CPAES
- [ ] Miembro de la CGE
- [ ] Miembro de la CSE
- [ ] Miembro de la CDA
- [ ] Miembro de la CMAP
- [ ] Personal de la Secretaría de la UICN
- [ ] Otros (especificar)

2. ¿En qué país / territorio está usted ubicado?

3. Género

- ○ Femenino
- ○ Masculino

* 4. ¿Con qué frecuencia debe consultar literatura científica para llevar a cabo su trabajo relacionado con la UICN?

Nunca	Raramente	A veces (una vez al mes)	A menudo (una vez a la semana)	Muy frecuentemente (a diario)
○	○	○	○	○

* 5. ¿Cuán fácil es para usted obtener la literatura científica que necesita para llevar a cabo su trabajo relacionado con la UICN?

No es nada fácil	No es fácil	Fácil	Muy fácil
○	○	○	○

Por favor, explique su respuesta (si lo considera necesario).

* 6. ¿Qué importancia tiene el acceder fácilmente la literatura científica para su trabajo con la UICN?

Nada importante	Algo importante	Muy importante	Esencial
○	○	○	○

Explique su respuesta si lo desea.

7. En general, ¿con qué frecuencia el empleado termina su trabajo en las fechas programadas de entrega?

- ○ Casi siempre
- ○ Frecuentemente
- ○ A veces
- ○ Rara vez
- ○ Casi nunca

8. ¿En qué formato prefiere leer literatura científica?

	Prefiero leer en una pantalla	Prefiero imprimir para leer	Prefiero el original impreso
Artículos en revistas científicas	◯	◯	◯
Libros	◯	◯	◯

Otros (por favor especifique)

* 9. ¿Tiene usted acceso institucional a literatura científica en línea (por ejemplo a través de su afiliación a una universidad u otra organización)?

◯ Si

◯ No

* 10. ¿Con qué frecuencia utiliza usted los siguientes medios para tener acceso a la literatura científica en su trabajo relacionado con la UICN?

	Nunca o no disponible	Con poca frecuencia	A veces (una vez al mes)	A menudo (una vez a la semana)	Muy frecuentemente (a diario)
Utilizo la biblioteca de mi propia institución.	○	○	○	○	○
Voy a una biblioteca local (pública, académica, etc.) para leer literatura científica impresa.	○	○	○	○	○
Consulto literatura científica en línea a través de un acceso institucional (por ejemplo a través de mi afiliación con una universidad u otra organización).	○	○	○	○	○
Solicito artículos de revistas de la Biblioteca de la UICN.	○	○	○	○	○
Solicito artículos de revistas directamente al autor.	○	○	○	○	○
Accedo a través de mi propia suscripción a título personal a una o varias revistas.	○	○	○	○	○
Le pregunto a un amigo o colega que tiene acceso a literatura científica en línea.	○	○	○	○	○
Accedo a todo lo que pueda encontrar en Internet de forma gratuita (a través de Google Scholar, revistas de acceso libre, ResearchGate, etc.)	○	○	○	○	○

11. Si tuviera acceso institucional a literatura científica en línea, con qué frecuencia la utilizaría para su trabajo con la UICN?

Nunca	Raramente	A veces (una vez al mes)	A menudo (una vez a la semana)	Muy frecuentemente (a diario)
○	○	○	○	○

* 12. ¿Qué efecto tendría el acceso institucional a literatura científica en línea sobre la calidad de su trabajo con la UICN?

Ningún efecto en absoluto	Un ligero efecto positivo	Efecto positivo moderado	Gran efecto positivo
○	○	○	○

Explique su respuesta si lo desea.

* 13. ¿Qué impacto tiene la falta de acceso institucional a literatura científica en línea sobre su trabajo con la UICN?

Ningún impacto en absoluto	Un leve impacto negativo	Un impacto negativo moderado	Un gran impacto negativo
○	○	○	○

Explique su respuesta si lo desea.

14. ¿Tiene algún otro comentario, preguntas o sugerencias?

15. Si no le importa ser contactado en el futuro (por ejemplo, para dar más detalles sobre sus respuestas o para participar en un grupo focal sobre la temática), por favor deje su información de contacto a continuación.

Nombre

Dirección de correo electrónico
