# Supplementary material for: Access to scientific literature by the conservation community
Source: PeerJ. 2020 Jul 9;8:e9404. doi: 10.7717/peerj.9404 (PMC7354838; doi:10.7717/peerj.9404)
Supplement: Supplemental Information 3 [file peerj-08-9404-s003.pdf]

Enquête de l'UICN sur l’accès à la littérature scientifique

Merci de répondre à cette enquête de la bibliothèque du siège de l’UICN au sujet de votre accès à la littérature scientifique

Le but de cette enquête est de comprendre l’importance de l’accès à la littérature scientifique pour le Secrétariat de l’UICN, les Commissions et les Membres afin de guider l’unité Science et Connaissance - et notamment la bibliothèque du siège de l’UICN - et améliorer notre soutien en la matière. Pour ce faire, nous avons besoin de comprendre quels sont les niveaux et moyens d’accés à la littérature scientifique dont vous précisez. Au cours de cette enquête, nous définissons le terme « littérature scientifique » comme les revues scientifiques évaluées par des pairs et les ouvrages techniques.

Cette enquête devrait vous prendre 5 à 7 minutes et est anonyme. Vous aurez la possibilité de laisser vos coordonnées à la fin si vous le souhaitez.

1. Quelle est votre relation avec l'UICN? Cochez tout ce qui correspond.

- ☐ Etat Membre
- ☐ Agence gouvernementale Membre
- ☐ ONG internationale Membre
- ☐ ONG nationale Membre
- ☐ Membre affilié
- ☐ Membre CEC
- ☐ Membre CPEES
- ☐ Membre CGE
- ☐ Membre CSE
- ☐ Membre CMDE
- ☐ Membre CMAP
- ☐ Personnel du Secrétariat
- ☐ Autres (veuillez spécifier)

2. Dans quel pays/territoire êtes-vous basé?

3. Sexe

- ○ Femme
- ○ Homme

* 4. A quelle fréquence devez-vous consulter de la littérature scientifique pour votre travail lié à l'UICN?

Jamais	Rarement	Parfois (une fois par mois)	Souvent (une fois par semaine)	Très fréquemment (tous les jours)
◯	◯	◯	◯	◯

* 5. Avec quelle facilité arrivez-vous à obtenir de la littérature scientifique dont vous avez besoin pour votre travail lié à l'UICN?

Difficilement	Pas facilement	Facilement	Très facilement
○	○	○	○

N'hésitez pas à fournir plus d'explications (si nécessaire)

* 6. Quelle importance a la facilité d'accès à la littérature scientifique pour votre travail lié à l'UICN?

Pas d'importance	Peu d'importance	Très important	Essentiel
○	○	○	○

N'hésitez pas à fournir plus d'explications (si nécessaire)

7. Quel est le journal scientifique qui aurait le plus d'impact sur votre travail lié à l'UICN si vous pouviez y accéder facilement?

8. Dans quel format préférez-vous lire la littérature scientifique?

	Je préfère lire sur écran	Je préfère imprimer pour lire	Je préfère lire l'exemplaire original en papier
Articles	○	○	○
Livres	○	○	○

N'hesitez pas à fournir plus d'exceptions (si besoin)

* 9. Avez-vous un accès institutionnel à la littérature scientifique en ligne (par exemple par affiliation à une université ou autre organisation)?

- Oui
- Non

* 10. Avec quelle fréquence utilisez-vous les moyens suivants pour accéder à la littérature scientifique pour votre travail lié à l'UICN?

	Jamais ou pas disponible	Rarement	Parfois (une fois par mois)	Souvent (une fois par semaine)	Très fréquemment (tous les jours)
J'utilise la bibliothèque de mon institution.	○	○	○	○	○
J'utilise une bibliothèque locale (publique, académique, etc.) pour lire des exemplaires de littérature scientifique en version imprimée.	○	○	○	○	○
J'ai un accès institutionnel à la littérature scientifique en ligne (par affiliation à une université ou autre organisation).	○	○	○	○	○
Je demande les articles à la bibliothécaire de l'UICN.	○	○	○	○	○
Je demande les articles directement à leur auteur.	○	○	○	○	○
J'utilise mon inscription à titre privé pour lire différents journaux scientifiques.	○	○	○	○	○
Je demande à un ami ou collègue qui a accès à la littérature scientifique en ligne.	○	○	○	○	○
J'utilise ce que je trouve en ligne et gratuitement (par exemple à travers Google Scholar, à travers de journaux en libre-accès, ResearchGate, etc.).	○	○	○	○	○

11. Si vous aviez un accès institutionnel à la littérature scientifique en ligne, avec quelle fréquence l'utiliseriez-vous pour votre travail lié à l'UICN?

Jamais	Rarement	Parfois (une fois par semaine)	Souvent (une fois par semaine)	Très fréquemment (tous les jours)
○	○	○	○	○

* 12. Quel impact aurait un accès institutionnel à la littérature scientifique en ligne sur la qualité de votre travail lié à l'UICN?

Pas d'effet	Effet positif faible	Effet positif modéré	Grand effet positif
○	○	○	○

N'hesitez pas à fournir plus d'explications (si besoin)

* 13. Quel impact a le manque d'accès institutionnel à la littérature scientifique en ligne sur votre travail lié à l'UICN?

Pas d'impact	Impact négatif faible	Impact négatif modéré	Grand impact négatif
○	○	○	○

N'hesitez pas à fournir plus d'explications (si besoin)

14. Avez-vous d'autres commentaires, suggestions ou questions?

15. Si vous ne voyez pas d'inconvénient à être contacté à l'avenir (par exemple pour développer vos réponses ou participer à des groupes de discussions ciblés sur cette thématique), merci de laisser vos coordonnées ci-dessous.

Nom

Courriel
