# Supplementary material for: Access to scientific literature by the conservation community
Source: PeerJ. 2020 Jul 9;8:e9404. doi: 10.7717/peerj.9404 (PMC7354838; doi:10.7717/peerj.9404)
Supplement: Supplemental Information 6 [file peerj-08-9404-s006.pdf]

```
## ---------------------------
##
## Script name: Process_Library_Survey_Data
##
## Purpose of script: fits a binomial logistic regresion model to the
library survey data.
##
## Author: Nicholas Macfarlane, PhD
##
## Date Created: 2020-04-10
##
##
## ---------------------------
##
## Notes:
##
##
## ---------------------------

## set working directory for PC

setwd("D:/Home/Desktop/IUCN/Library Survey/working")


## ---------------------------

## load packages:

require(multcomp)


## ---------------------------
## read in cleaned and arranged libary survey data file
dat<- read.csv("libraryfrequencyworking_for_analysis.csv", header=T) #1970
x 15 data table of survey responses

#head(dat)
# RespondentID AccessEase Language Gender Region InstitutionalAccess
IUCNCECmember IUCNCEESPmember
# 1   4861786455          1       EN      M      2                   0
1             0
# 2   4862370473          1       EN      F      5                   0
0             0
# 3   4862499333          1       EN      M      5                   0
0             0
# 4   4862762458          1       EN      F      5                   0
0             0
# 5   4863451450          1       EN      M      2                   1
0             0
# 6   4866173330          1       EN      M      5                   0
0             0
# IUCNCEMmember IUCNSSCmember IUCNWCELmember IUCNWCPAmember
IUCNSecretariatstaff    Sector Discipline
# 1             0             0              0              0
0    NatNGO        CEC
# 2             0             0              0              1
0 GovAgency       WCPA
```

```
# 3             0             0              0              1
0    NatNGO       WCPA
# 4             0             0              0              1
0    NatNGO       WCPA
# 5             0             1              0              0
0    NatNGO        SSC
# 6             0             0              0              1
0    IntNGO       WCPA


## ---------------------------

## input data processing

# create binary vector for insitutional access ease where 1 is easy or very easy and 0 is hard or very hard
dat$binary <- rep(0, length(dat$AcessEase))
for (i in 1:length(dat$AccessEase)){
  if(dat$AccessEase[i]>= 3)
    dat$binary[i] <- 1
  else dat$binary[i] <-0
}


# process data as factors
dat$AccessEase <-as.factor(dat$AccessEase)
dat$Language <-as.factor(dat$Language)
dat$Gender <-as.factor(dat$Gender)
dat$Region <-as.factor(dat$Region)
dat$InstitutionalAccess <-as.factor(dat$InstitutionalAccess)

## -----------------------------

## model selection and analysis.

## binomial logistic regression model fitting using AIC scores
m1 =glm(binary~ InstitutionalAccess+ Language+ Gender+ Sector+ Region+ Discipline, family="binomial", data = dat)
m2 =glm(binary~ InstitutionalAccess+ Language+ Gender+ Sector+ Region+ IUCNCECmember+ IUCNCEESPmember+ IUCNCEMmember + IUCNSSCmember + IUCNWCELmember + IUCNWCPAmember, family="binomial", data = dat)
m3 =glm(binary~ InstitutionalAccess+ Language+ Gender+ Region+ IUCNCECmember+ IUCNCEESPmember+ IUCNCEMmember + IUCNSSCmember + IUCNWCELmember + IUCNWCPAmember, family="binomial", data = dat)

m4 =glm(binary~ InstitutionalAccess+ Gender+ Region+ IUCNCECmember+ IUCNCEESPmember+ IUCNCEMmember + IUCNSSCmember + IUCNWCELmember + IUCNWCPAmember, family="binomial", data = dat)
m5 =glm(binary~ InstitutionalAccess+ Gender+ Region, family="binomial", data = dat)

## kruskal wallis comparisons
kruskal.test(binary~Language, data=dat)
kruskal.test(binary~Discipline, data=dat)
kruskal.test(binary~Region, data=dat)

#interactions
```

```
m5a =glm(binary~ InstitutionalAccess*Gender+ Region, family="binomial",
data = dat)
m5b =glm(binary~ InstitutionalAccess+Gender*Region, family="binomial", data
= dat)
m5c =glm(binary~ InstitutionalAccess*Region + Gender, family="binomial",
data = dat)

# tukey post hoc contrasts
t5<-glht(m5, linfct = mcp(Region = "Tukey"))
```
